# Assessment of the Risk of Venous Thromboembolism in Nonhospitalized Patients With COVID-19

**DOI:** 10.1001/jamanetworkopen.2023.2338

**Published:** 2023-03-13

**Authors:** Margaret C. Fang, Kristi Reynolds, Grace H. Tabada, Priya A. Prasad, Sue Hee Sung, Anna L. Parks, Elisha Garcia, Cecilia Portugal, Dongjie Fan, Ashok P. Pai, Alan S. Go

**Affiliations:** 1 Division of Hospital Medicine, The University of California, San Francisco; 2 Department of Research and Evaluation, Kaiser Permanente Southern California, Pasadena; 3 Department of Health System Science, Kaiser Permanente Bernard J. Tyson School of Medicine, Pasadena, California; 4 Division of Research, Kaiser Permanente Northern California, Oakland; 5 Division of Hematology/Oncology, University of Utah, Salt Lake City; 6 Department of Hematology/Oncology, Kaiser Permanente Oakland Medical Center, Oakland, California; 7 Department of Medicine, University of California, San Francisco; 8 Department of Epidemiology and Biostatistics, University of California, San Francisco; 9 Department of Medicine, Stanford University, Palo Alto, California

## Abstract

**Importance:**

Patients hospitalized with COVID-19 have higher rates of venous thromboembolism (VTE), but the risk and predictors of VTE among individuals with less severe COVID-19 managed in outpatient settings are less well understood.

**Objectives:**

To assess the risk of VTE among outpatients with COVID-19 and identify independent predictors of VTE.

**Design, Setting, and Participants:**

A retrospective cohort study was conducted at 2 integrated health care delivery systems in Northern and Southern California. Data for this study were obtained from the Kaiser Permanente Virtual Data Warehouse and electronic health records. Participants included nonhospitalized adults aged 18 years or older with COVID-19 diagnosed between January 1, 2020, and January 31, 2021, with follow-up through February 28, 2021.

**Exposures:**

Patient demographic and clinical characteristics identified from integrated electronic health records.

**Main Outcomes and Measures:**

The primary outcome was the rate per 100 person-years of diagnosed VTE, which was identified using an algorithm based on encounter diagnosis codes and natural language processing. Multivariable regression using a Fine-Gray subdistribution hazard model was used to identify variables independently associated with VTE risk. Multiple imputation was used to address missing data.

**Results:**

A total of 398 530 outpatients with COVID-19 were identified. The mean (SD) age was 43.8 (15.8) years, 53.7% were women, and 54.3% were of self-reported Hispanic ethnicity. There were 292 (0.1%) VTE events identified over the follow-up period, for an overall rate of 0.26 (95% CI, 0.24-0.30) per 100 person-years. The sharpest increase in VTE risk was observed during the first 30 days after COVID-19 diagnosis (unadjusted rate, 0.58; 95% CI, 0.51-0.67 per 100 person-years vs 0.09; 95% CI, 0.08-0.11 per 100 person-years after 30 days). In multivariable models, the following variables were associated with a higher risk for VTE in the setting of nonhospitalized COVID-19: age 55 to 64 years (HR 1.85 [95% CI, 1.26-2.72]), 65 to 74 years (3.43 [95% CI, 2.18-5.39]), 75 to 84 years (5.46 [95% CI, 3.20-9.34]), greater than or equal to 85 years (6.51 [95% CI, 3.05-13.86]), male gender (1.49 [95% CI, 1.15-1.96]), prior VTE (7.49 [95% CI, 4.29-13.07]), thrombophilia (2.52 [95% CI, 1.04-6.14]), inflammatory bowel disease (2.43 [95% CI, 1.02-5.80]), body mass index 30.0-39.9 (1.57 [95% CI, 1.06-2.34]), and body mass index greater than or equal to 40.0 (3.07 [1.95-4.83]).

**Conclusions and Relevance:**

In this cohort study of outpatients with COVID-19, the absolute risk of VTE was low. Several patient-level factors were associated with higher VTE risk; these findings may help identify subsets of patients with COVID-19 who may benefit from more intensive surveillance or VTE preventive strategies.

## Key Points

**Question:**

What is the risk of venous thromboembolism (VTE) among outpatients with COVID-19?

**Findings:**

In this cohort study of 398 530 adult outpatients with COVID-19, the rate of VTE was low in the first 30 days after COVID-19 diagnosis and even lower after 30 days of follow-up. Factors associated with a higher risk of VTE in COVID-19 included age 55 years or older, being male, a history of VTE or thrombophilia, and body mass index greater than or equal to 30.0.

**Meaning:**

The findings of this study suggest that the overall risk of VTE among outpatients with COVID-19 is low, but higher in the first 30 days after diagnosis.

## Teaser

This cohort study examines the risk of venous thromboembolism in patients with COVID-19 who are not hospitalized.

## Introduction

As of February 2023, more than 754 million confirmed cases of COVID-19 have occurred worldwide.^1^ Venous thromboembolism (VTE) has been recognized as an important complication of COVID-19.^2^ Most studies have focused on VTE outcomes occurring among hospitalized or critically ill patients with COVID-19 and have described a substantially increased risk compared with hospitalized patients without COVID-19.^3^ Therapeutically dosed anticoagulation may benefit selected hospitalized patients with COVID-19 and prophylactically dosed anticoagulants are recommended for all other hospitalized patients with COVID-19.^4,5,6,7^ However the vast majority of people with COVID-19 have milder disease and do not require hospitalization. Whether nonhospitalized (ie, outpatient) adults with COVID-19 have a clinically important risk of VTE has not been well elucidated. The relatively few studies that reported rates of VTE in outpatients with COVID-19 have conflicting results, with some reporting rates as low as 1.8 per 1000 persons tested (comparable to rates in the general population)^8,9,10^ while rates as high as 18% of ambulatory emergency department patients with COVID-19 have been observed.^11,12^ Precise estimates of VTE rates in outpatients with COVID-19 have also been limited by relatively small sample sizes or use of administrative codes to identify VTE that, when used alone, have poor accuracy to identify true VTE events.^13,14^ A recent clinical trial investigating prehospitalization prophylaxis against VTE in COVID-19 was terminated early because of a lower-than-anticipated event rate, demonstrating the challenges to prospectively study VTE in outpatients with COVID-19.^15^ Accurate assessments of the true risk of VTE are necessary to guide recommendations on prevention and surveillance strategies.

We conducted a large observational cohort study of adults with COVID-19 who did not require hospitalization and quantified the risk of incident VTE after COVID-19 diagnosis. Using comprehensive clinical databases from 2 integrated health care delivery systems managing a large ambulatory patient population, we hoped our study could provide more precise estimates of the true rates of VTE in outpatients with COVID-19. We also evaluated patient-level factors that may be associated with differential VTE risk, hypothesizing that specific patient characteristics, such as age or a history of VTE, are associated with a greater risk for VTE in people with COVID-19.

## Methods

### Population and Setting

This was a retrospective cohort study of adults aged 18 years or older enrolled in Kaiser Permanente Northern California (KPNC) and Kaiser Permanente Southern California (KPSC), 2 large integrated health care delivery systems providing comprehensive outpatient, emergency, and inpatient care for approximately 9 million health plan members across California (>4.5 million through KPNC and >4.6 million through KPSC). The membership is highly representative of the local surrounding and California statewide population regarding age, gender, and self-reported race and ethnicity.^16,17^ This study was approved by the KPNC and KPSC institutional review boards, and a waiver of informed consent was obtained due to the nature of the study. We followed the Strengthening the Reporting of Observational Studies in Epidemiology (STROBE) reporting guideline for cohort studies.

Data for this study were obtained from the Kaiser Permanente Virtual Data Warehouse and electronic health records (EHRs).^18,19^ The Virtual Data Warehouse contains linked EHR-based data tables that include information about patient demographic characteristics, health service use and encounter data, pharmacy dispensing, and laboratory and radiology test results.^20^

### Population at Risk

We first identified all adults in the source populations with laboratory-confirmed COVID-19 based on a nasopharyngeal or oropharyngeal polymerase chain reaction test for SARS-CoV-2 virus obtained between January 1, 2020, and January 31, 2021. Test results obtained internally and from outside laboratories that used approved assays were available in participating sites’ laboratory systems. The study period encompassed the first 2 waves of COVID-19 and was therefore performed before the widespread availability of COVID-19 vaccines and home-based rapid antigen testing. As our focus was on patients who did not require hospitalization for COVID-19, we only included those with tests performed in outpatient settings and who were not hospitalized as part of the initial clinical encounter. If patients had multiple test results, the date of the first positive test was considered the index COVID-19 diagnosis date. Patients with missing data on age or gender or who had less than 6 months of continuous health plan enrollment or pharmacy benefits before the index date were excluded to ensure systematic capture of baseline characteristics and medical conditions.

### Outcomes

Follow-up for all patients in the cohort continued until February 28, 2021, with censoring by death, health plan disenrollment, or hospitalization attributed to COVID-19 (defined as a hospitalization within 30 days of their index COVID-19 test date that was unrelated to VTE). Death was identified based on comprehensive data from health plan databases (including inpatient and emergency department deaths and proxy reports of deaths), California state death certificate files, and the Social Security Administration Death Master File.

The primary outcome was diagnosis of a new, acute VTE event after the index positive COVID-19 test date. We first identified possible VTE events by searching for outpatient, emergency department, and inpatient clinical encounters that had *International Statistical Classification of Diseases and Related Health Problems, 10th Revision* (*ICD-10*) diagnosis codes for VTE (excluding codes for superficial venous thromboses and pregnancy-related VTE) (eTable in Supplement 1). To increase the likelihood that a particular outpatient encounter was for a new VTE event, we restricted the data to encounters in which a relevant radiologic test to diagnose VTE, such as chest computed tomography angiogram or extremity ultrasonography, was obtained within 14 days before or after the clinical encounter. Although patients were censored at the time of hospitalization if hospitalized within 30 days of the index outpatient COVID-19 diagnosis, we acknowledged some patients with VTE may require hospitalization for the VTE itself. Thus, for patients with a hospital diagnosis of VTE within 30 days, we included these events as possible outcomes only if there was a confirmatory radiologic procedure for VTE performed on or before the hospital admission date. Venous thromboembolism events without a relevant radiologic procedure or procedures occurring after the admission date were excluded, as these events were considered potentially related to the hospitalization and not to the outpatient COVID-19 diagnosis.

Once patients with potential VTE events were identified, we applied an internally developed and validated natural language processing algorithm to further determine which encounters represented true acute VTE events. This algorithm was trained on 479 physician-confirmed VTE events and had a positive predictive value of 95% and a negative predictive value of 97% compared with physician adjudication of medical records using standardized case review criteria.^21,22^ Confirmed VTE events were categorized as pulmonary embolism (with or without other concomitant deep venous thromboses [DVT]), lower extremity DVTs, upper extremity DVTs, and other thromboses of unusual sites (eg, splanchnic, retinal vein, or cerebral venous sinus).

### Covariates

Sociodemographic information (age, self-reported gender, and self-reported race and Hispanic ethnicity) and baseline medical and pharmacy information were obtained from EHR data. Race and ethnicity were reported to provide additional context about the population included in this study. Available categories of race were Alaska Native, Asian or Pacific Islander, Black, Multiple, Native American, and White. Ethnicity was available as Hispanic or non-Hispanic. Baseline medical conditions relevant to VTE risk were identified by searching the EHR data for relevant inpatient or outpatient *International Classification of Diseases, 9th Revision* or *ICD-10* codes within 5 years before or on the index date. Risk factors for VTE included diagnosed thrombophilia (defined as *ICD* codes for “primary hypercoagulable state” and “secondary hypercoagulable state”), hypercoagulable hematologic conditions, and cancer. We were not able to distinguish between active and inactive cancer. Kidney function was determined using the Chronic Kidney Disease Epidemiology Collaboration–estimated glomerular filtration rate equation using outpatient serum creatinine values found using EHR data up to 3 years before or on the index date.^23^ Similarly, outpatient hemoglobin and body mass index (calculated as weight in kilograms divided by height in meters squared) levels were obtained from EHR data up to 3 years before or on the index date.

Health plan outpatient pharmacy dispensing records were used to identify baseline use of oral anticoagulants (defined as an active prescription for warfarin, dabigatran, rivaroxaban, apixaban, or edoxaban within 60 days before the index date) based on data on estimated days supplied per dispensed prescription and refill patterns in pharmacy databases using validated methods.^24^ For risk adjustment purposes, we also identified baseline use of other cardiovascular medications (ie, angiotensin-converting enzyme inhibitors, angiotensin II receptor blockers, β-blockers, calcium channel blockers, diuretics, aldosterone receptor antagonists, nonaspirin antiplatelet agents, and lipid-lowering agents) up to 120 days before the index date. Aspirin was not included because it was available without prescription, so we were unable to reliably capture its use.

### Statistical Analysis

Analyses were performed using SAS, version 9.4 (SAS Institute LLC). Baseline characteristics are presented as means (SDs) or medians (IQRs) for continuous variables and frequencies with percentages for categorical variables.

We plotted the cumulative incidence of VTE, accounting for the competing risk for death or health plan disenrollment. We also calculated crude rates (per 100 person-years) of VTE with associated 95% CIs overall and in subgroups of patients previously reported to have higher VTE risk in other settings (ie, men, prior VTE, thrombophilia, cancer, and high body mass index).^25^ In addition, we identified multivariable predictors of VTE in the setting of outpatient COVID-19 using a Fine-Gray subdistribution hazard model to account for competing risks of death or health care plan disenrollment and including all available baseline variables as candidate predictors. We used a multiple imputation approach across 30 imputed data sets to account for missing data in laboratory variables. With 2-sided, unpaired testing, the significance threshold was 0.05.

## Results

**Figure 1.**
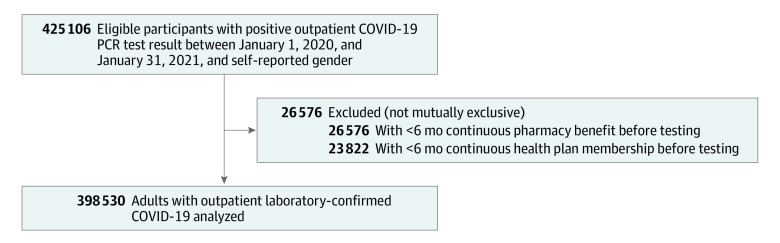
Cohort of Adults With Outpatient COVID-19 PCR indicates polymerase chain reaction.

Between January 1, 2020, and January 31, 2021, we identified 398 530 adults with outpatient laboratory-confirmed COVID-19 who met study eligibility criteria (Figure 1). Mean (SD) age was 43.8 (15.8) years, 53.7% were women, 10.6% were Asian or Pacific Islander, 6.1% were Black, 75.7% were White, and 54.3% were of self-reported Hispanic ethnicity (Table 1). Few of the nonhospitalized patients in our study had a documented prior VTE (0.3% of the cohort) or baseline oral anticoagulant use (1.1%).

**Table 1.** Baseline Characteristics of Outpatient Adults With Laboratory-Confirmed COVID-19 Infection Between January 1, 2020, and January 31, 2021

Baseline characteristic	Overall, No. (%)
No.	398 530
Age, mean (SD) [range]	43.8 (15.8) [18.0-105.9]
Age group, y	
≤54	296 454 (74.4)
55-64	61 186 (15.4)
65-74	28 640 (7.2)
75-84	9553 (2.4)
≥85	2697 (0.7)
Gender	
Women	214 150 (53.7)
Men	184 380 (46.3)
Race	
Asian or Pacific Islander	42 078 (10.6)
Black	24 376 (6.1)
White	301 852 (75.7)
Other^a^	5560 (1.4)
Unknown	24 664 (6.2)
Hispanic ethnicity	216 232 (54.3)
Baseline medical history	
Cardiovascular diseases	
Coronary heart disease	5069 (1.3)
Acute myocardial infarction	1599 (0.4)
Unstable angina	702 (0.2)
Percutaneous coronary intervention	4024 (1.0)
Coronary artery bypass surgery	590 (0.1)
Heart failure	3273 (0.8)
Rheumatic heart disease	699 (0.2)
Atrial fibrillation or flutter	6044 (1.5)
Mitral and/or aortic valvular disease	3533 (0.9)
Peripheral artery disease	2782 (0.7)
Ischemic stroke or transient ischemic attack	2508 (0.6)
Cardiovascular risk factors	
Diabetes	54 112 (13.6)
Hypertension	86 048 (21.6)
Dyslipidemia	123 189 (30.9)
Former or current smoker	90 259 (22.6)
Risk factors for thrombosis	
Prior VTE	1279 (0.3)
Thrombophilia^b^	915 (0.2)
Hypercoagulable hematologic conditions^c^	1794 (0.5)
Hemiplegia or paraplegia	1352 (0.3)
Inflammatory bowel disease	2006 (0.5)
Rheumatic disease	3828 (1.0)
Cancer	7644 (1.9)
Other medical history	
Coagulopathy	5937 (1.5)
Chronic lung disease	49 604 (12.4)
Chronic liver disease	17 363 (4.4)
Moderate or severe liver disease	1933 (0.5)
Mild liver disease	1734 (0.4)
Peptic ulcer disease	697 (0.2)
Hospitalization for gastrointestinal hemorrhage	788 (0.2)
Intracranial hemorrhage	
Nontraumatic	672 (0.2)
Traumatic	441 (0.1)
Hospitalization for other bleeding	173 (0.0)
Prior mechanical fall	2050 (0.5)
Diagnosed dementia	1914 (0.5)
Diagnosed depression	48 883 (12.3)
Substance abuse	5185 (1.3)
Alcohol abuse	10 372 (2.6)
Anticoagulant within 60 d before index date	4360 (1.1)
Baseline medication use	
Angiotensin-converting enzyme inhibitor	35 327 (8.9)
Angiotensin II receptor blocker	19 779 (5.0)
β-Blocker	25 503 (6.4)
Calcium channel blocker	21 186 (5.3)
Diuretic	33 169 (8.3)
Aldosterone receptor antagonist	2565 (0.6)
Nonaspirin antiplatelet agent	2769 (0.7)
Statin	60 894 (15.3)
Other lipid-lowering agents	2624 (0.7)
Vital signs	
Systolic blood pressure, mm Hg	
≥180	457 (0.1)
160-179	2925 (0.7)
140-159	23 412 (5.9)
130-139	94 353 (23.7)
121-129	89 720 (22.5)
≤120	154 225 (38.7)
Missing	33 438 (8.4)
Diastolic blood pressure, mm Hg	
≥110	282 (0.1)
100-109	1599 (0.4)
90-99	9679 (2.4)
85-89	29 849 (7.5)
81-84	32 850 (8.2)
≤80	290 810 (73.0)
Missing	33 461 (8.4)
BMI	
<18.5	2848 (0.7)
18.5-24.9	69 915 (17.5)
25.0-29.9	116 297 (29.2)
30.0-39.9	137 604 (34.5)
≥40	34 303 (8.6)
Missing	37 563 (9.4)
Laboratory values	
Estimated glomerular filtration rate, mL/min/1.73 m^2^	
≥90	178 769 (44.9)
60-89	77 834 (19.5)
45-59	8142 (2.0)
30-44	2556 (0.6)
15-29	830 (0.2)
<15	673 (0.2)
Dialysis or transplant	574 (0.1)
Missing	129 152 (32.4)
Serum creatinine, mg/dL	
Mean (SD)	0.9 (0.5)
Median (IQR)	0.8 (0.7-1.0)
Missing	129 152 (32.4)
Hemoglobin, g/dL	
≥13.0	190 962 (47.9)
12.0-12.9	43 802 (11.0)
11.0-11.9	18 182 (4.6)
10.0-10.9	6860 (1.7)
9.0-9.9	2606 (0.7)
<9.0	1399 (0.4)
Missing	134 719 (33.8)

Abbreviations: BMI, body mass index (calculated as weight in kilograms divided by height in meters squared); VTE, venous thromboembolism.

SI conversion factors: To convert creatinine to micromoles per liter, multiply by 88.4; hemoglobin to grams per liter, multiply by 10.

^a^ Other races included Native American, Alaska native, or multiple races.

^b^ Thrombophilia: *International Classification of Diseases, 9th Revision* (*ICD*-9) or *International Statistical Classification of Diseases and Related Health Problems, 10th Revision* (*ICD*-*10*) diagnosis of primary or secondary hypercoagulable state.

^c^ Hypercoagulable hematologic conditions: *ICD-9* or *ICD-10* diagnoses of familial polycythemia, polycythemia vera, hemoglobinuria due to hemolysis from external causes, disturbances of sulfur-bearing amino-acid metabolism, congenital deficiency of other clotting factors, defibrination syndrome, essential thrombocythemia, myelofibrosis with myeloid metaplasia, and monoclonal paraproteinemia.

**Figure 2.**
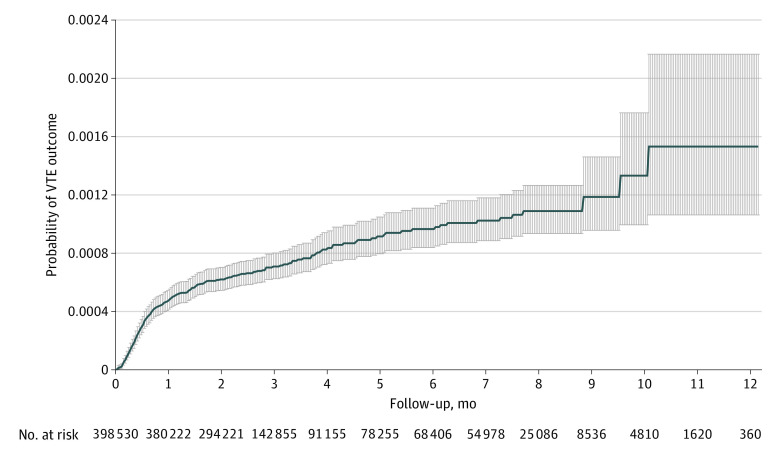
Cumulative Incidence of Venous Thromboembolism (VTE) Adjusted for competing risks of death or disenrollment among adult outpatients with confirmed COVID-19 infection from January 1, 2020, through January 31, 2021. Solid line indicates rate; shaded area, 95% CI.

During follow-up, we identified 292 confirmed VTE events, for an overall rate of 0.26 (95% CI, 0.24-0.30) per 100 person-years. Among the 292 VTE events, 57.5% were pulmonary emboli, 34.9% were lower extremity DVTs, 5.1% were upper extremity DVTs, and 2.5% were other VTEs at unusual sites. During the first 12 months of follow-up, after accounting for potential competing risks for death or health care plan disenrollment, the sharpest increase in cumulative incidence of VTE risk was observed during the first 30 days after laboratory-confirmed COVID-19 (Figure 2). The crude rate of VTE was 0.58 (95% CI, 0.51-0.67) per 100 person-years in the first 30 days and 0.09 (95% CI, 0.08-0.11) per 100 person-years after 30 days. The median time between index COVID-19 diagnosis and date of VTE event was 19.0 (IQR, 10.5-45.0) days. In unadjusted analyses, age 55 years or older, male gender, prior VTE, known thrombophilia, and body mass index greater than or equal to 30.0 were all associated with higher rates of VTE (Table 2).

**Table 2.** Unadjusted Rates of VTE Among Subgroups of Adults With Confirmed Outpatient COVID-19 Infection

Variable	Overall VTE after COVID-19 diagnosis		VTE occurring within 30 d of index COVID-19 diagnosis		VTE occurring >30 d after index COVID-19 diagnosis	
	No. (%)	Rate per 100 person-years follow-up (95% CI)	No. (%)	Rate per 100 person-years follow-up (95% CI)	No. (%)	Rate per 100 person-years follow-up (95% CI)
Overall	292 (0.1)	0.26 (0.24-0.30)	191 (0.05)	0.58 (0.51-0.67)	101 (0.03)	0.09 (0.08-0.11)
Age group, y						
≤54	116 (0.04)	0.14 (0.11-0.17)	77 (0.03)	0.31 (0.25-0.39)	39 (0.01)	0.05 (0.03-0.06)
55-64	62 (90.1)	0.38 (0.30-0.49)	43 (0.1)	0.87 (0.64-1.17)	19 (0.03)	0.12 (0.07-0.18)
65-74	64 (0.2)	0.91 (0.71-1.16)	36 (0.1)	1.61 (1.16-2.23)	28 (0.1)	0.40 (0.28-0.58)
75-84	37 (0.4)	1.75 (1.27-2.42)	28 (0.3)	3.96 (2.73-5.73)	9 (0.1)	0.43 (0.22-0.82)
≥85	13 (0.5)	2.30 (1.33-3.96)	7 (0.3)	3.80 (1.81-7.97)	6 (0.2)	1.06 (0.48-2.36)
Gender						
Women	138 (0.1)	0.23 (0.20-0.27)	81 (0.04)	0.46 (0.37-0.57)	57 (0.03)	0.10 (0.07-0.12)
Men	154 (0.1)	0.30 (0.26-0.36)	110 (0.1)	0.73 (0.61-0.88)	44 (0.02)	0.09 (0.06-0.12)
Medical history						
Thrombophilia^a^	6 (0.7)	2.65 (1.19-5.89)	3 (0.3)	4.16 (1.34-12.91)	3 (0.3)	1.32 (0.43-4.11)
No thrombophilia	286 (0.1)	0.26 (0.23-0.29)	188 (0.1)	0.57 (0.50-0.66)	98 (0.02)	0.09 (0.07-0.11)
VTE	24 (1.9)	7.51 (5.04-11.21)	12 (0.9)	12.44 (7.06-21.90)	12 (0.9)	3.76 (2.13-6.61)
No VTE	268 (0.1)	0.24 (0.22-0.27)	179 (0.1)	0.55 (0.47-0.63)	89 (0.02)	0.08 (0.07-0.10)
BMI						
<18.5	1 (0.04)	0.13 (0.02-0.94)	0	0	1 (0.04)	0.13 (0.02-0.94)
18.5-24.9	35 (0.1)	0.18 (0.13-0.25)	23 (0.03)	0.40 (0.26-0.6)	12 (0.02)	0.06 (0.04-0.11)
25.0-29.9	71 (0.1)	0.22 (0.17-0.28)	48 (0.04)	0.50 (0.38-0.66)	23 (0.02)	0.07 (0.05-0.11)
30.0-39.9	122 (0.1)	0.32 (0.27-0.38)	75 (0.1)	0.67 (0.53-0.84)	47 (0.03)	0.12 (0.09-0.16)
≥40.0	50 (0.2)	0.54 (0.41-0.71)	35 (0.1)	1.27 (0.91-1.77)	15 (0.04)	0.16 (0.10-0.27)
Unknown	13 (0.03)	0.13 (0.07-0.22)	10 (0.03)	0.32 (0.17-0.59)	3 (0.01)	0.03 (0.01-0.09)

Abbreviations: BMI, body mass index (calculated as weight in kilograms divided by height in meters squared); VTE, venous thromboembolism.

^a^ Thrombophilia: *International Classification of Diseases, 9th Revision* or *International Statistical Classification of Diseases and Related Health Problems, 10th Revision* diagnosis of primary or secondary hypercoagulable state.

In multivariable models adjusted for baseline medical conditions and accounting for potential competing risks of death or health plan disenrollment, characteristics that were independently associated with a higher risk of VTE included age 55 to 64 years (adjusted HR, 1.85 [95% CI, 1.26-2.72]), 65 to 74 years (adjusted HR, 3.43 [95% CI, 2.18-5.39]), 75 to 84 years (adjusted HR, 5.46 [95% CI, 3.20-9.34]), greater than or equal to 85 years (adjusted HR, 6.51 [95% CI, 3.05-13.86]), male gender (adjusted HR, 1.49 [95% CI, 1.15-1.96]), prior VTE (adjusted HR, 7.49 [95% CI, 4.29-13.07]), thrombophilia (adjusted HR, 2.52 [95% CI, 1.04-6.14]), inflammatory bowel disease (adjusted HR, 2.43 [95% CI, 1.02-5.80]), body mass index 30.0 to 39.9 (adjusted HR, 1.57 [95% CI, 1.06-2.34]), and body mass index greater than or equal to 40.0 (adjusted HR, 3.07 [95% CI, 1.95-4.83]).(Table 3).

**Table 3.** Multivariable Associations of Time to First VTE, Adjusted for Competing Risk of Death or Medical Plan Disenrollment, Among Adults With Outpatient Laboratory-Confirmed COVID-19 Between January 1, 2020, and January 31, 2021

Characteristic	aHR (95% CI)^a^		
	Overall	Within 30 d of index COVID-19 diagnosis	>30 d after index COVID-19 diagnosis
Age, y			
≤54	1 [Reference]	1 [Reference]	1 [Reference]
55-64	1.85 (1.26-2.72)	1.96 (1.20-3.19)	1.62 (0.89-2.96)
65-74	3.43 (2.18-5.39)	3.08 (1.70-5.58)	3.90 (1.97-7.73)
75-84	5.46 (3.20-9.34)	7.05 (3.65-13.65)	2.99 (1.21-7.37)
≥85	6.51 (3.05-13.86)	6.41 (2.36-17.45)	6.50 (2.05-20.62)
Gender			
Women	1 [Reference]	1 [Reference]	1 [Reference]
Men	1.49 (1.15-1.96)	1.93 (1.37-2.70)	0.91 (0.59-1.41)
Race			
Asian or Pacific Islander	0.65 (0.39-1.09)	0.70 (0.37-1.31)	0.56 (0.23-1.36)
Black	1.43 (0.96-2.12)	1.96 (1.23-3.11)	0.69 (0.30-1.55)
White	1 [Reference]	1 [Reference]	1 [Reference]
Other	1.79 (0.91-3.54)	1.91 (0.83-4.41)	1.59 (0.49-5.19)
Unknown	0.24 (0.07-0.75)	0.13 (0.02-0.91)	0.43 (0.10-1.85)
Hispanic ethnicity	0.82 (0.62-1.10)	0.97 (0.68-1.37)	0.61 (0.38-1.01)
Baseline medical history			
Cardiovascular diseases			
Acute myocardial infarction	1.73 (0.61-4.93)	3.28 (0.93-11.53)	0.47 (0.07-3.08)
Unstable angina	0.60 (0.07-4.80)	1.08 (0.13-9.13)	NA
Percutaneous coronary intervention	1.24 (0.58-2.65)	0.76 (0.24-2.38)	1.79 (0.63-5.07)
Heart failure	0.54 (0.23-1.25)	0.63 (0.19-2.07)	0.47 (0.14-1.53)
Rheumatic heart disease	0.66 (0.12-3.56)	0.76 (0.08-7.45)	0.58 (0.05-6.81)
Atrial fibrillation or flutter	0.99 (0.50-1.94)	0.61 (0.23-1.60)	1.66 (0.64-4.32)
Mitral and/or aortic valvular disease	1.33 (0.68-2.63)	1.38 (0.54-3.50)	1.43 (0.53-3.86)
Peripheral artery disease	0.76 (0.32-1.82)	0.72 (0.22-2.39)	0.85 (0.22-3.20)
Ischemic stroke or transient ischemic attack	0.85 (0.31-2.30)	0.52 (0.11-2.44)	1.00 (0.23-4.37)
Cardiovascular risk factors			
Diabetes	1.01 (0.72-1.41)	0.80 (0.53-1.21)	1.48 (0.82-2.68)
Hypertension	1.12 (0.76-1.67)	1.23 (0.77-1.98)	0.92 (0.46-1.84)
Dyslipidemia	1.41 (1.02-1.95)	1.65 (1.10-2.46)	1.05 (0.61-1.81)
Former or current smoker	1.05 (0.81-1.37)	0.89 (0.64-1.24)	1.40 (0.89-2.19)
Risk factors for thrombosis			
Prior VTE	7.49 (4.29-13.07)	6.64 (2.76-15.95)	8.18 (3.92-17.08)
Thrombophilia	2.52 (1.04-6.14)	2.67 (0.79-9.05)	2.48 (0.59-10.35)
Hypercoagulable hematologic conditions	0.70 (0.20-2.46)	0.44 (0.06-3.17)	0.94 (0.14-6.43)
Hemiplegia or paraplegia	0.52 (0.12-2.19)	NA	1.00 (0.16-6.06)
Rheumatic disease	1.63 (0.80-3.34)	1.66 (0.65-4.23)	1.44 (0.41-5.02)
Cancer	1.49 (0.94-2.37)	1.31 (0.71-2.45)	1.86 (0.88-3.93)
Other medical history			
Coagulopathy	1.53 (0.92-2.54)	1.05 (0.47-2.38)	2.21 (1.09-4.49)
Chronic lung disease	1.15 (0.85-1.56)	0.95 (0.64-1.42)	1.56 (0.96-2.54)
Chronic liver disease	1.05 (0.64-1.72)	1.06 (0.57-1.99)	1.00 (0.45-2.23)
Moderate or severe liver disease	1.57 (0.37-6.56)	2.33 (0.33-16.71)	0.71 (0.37-1.38)
Mild liver disease	1.31 (0.30-5.69)	0.67 (0.08-5.66)	4.05 (1.69-9.73)
Peptic ulcer	2.24 (0.75-6.68)	2.32 (0.59-9.09)	2.19 (0.35-13.77)
Hospitalization for gastrointestinal hemorrhage	0.76 (0.24-2.36)	0.36 (0.05-2.43)	1.24 (0.25-6.11)
Intracranial hemorrhage, nontraumatic	2.09 (0.63-6.88)	1.00 (0.11-8.78)	2.02 (0.50-8.11)
Intracranial hemorrhage, traumatic	0.59 (0.09-3.98)	NA	1.33 (0.23-7.61)
Hospitalization for other bleeding	3.07 (0.60-15.65)	8.21 (1.71-39.33)	NA
Inflammatory bowel disease	2.43 (1.02-5.80)	3.46 (1.31-9.13)	1.05 (0.13-8.46)
Mechanical fall	1.02 (0.42-2.47)	0.67 (0.14-3.21)	1.21 (0.37-3.91)
Diagnosed dementia	1.22 (0.54-2.74)	1.87 (0.75-4.69)	0.51 (0.10-2.76)
Diagnosed depression	0.94 (0.68-1.30)	1.02 (0.67-1.55)	0.79 (0.46-1.34)
Substance abuse	1.47 (0.73-2.97)	1.36 (0.52-3.58)	1.84 (0.64-5.27)
Alcohol abuse	0.95 (0.54-1.69)	1.14 (0.57-2.26)	0.66 (0.25-1.77)
Vital signs and laboratory values			
BMI			
18.5-25.0	1 [Reference]	1 [Reference]	1 [Reference]
<18.5	0.52 (0.07-3.90)	N/A	1.21 (0.13-11.01)
25.0-29.9	1.06 (0.70-1.60)	1.07 (0.64-1.78)	1.06 (0.52-2.17)
30.0-39.9	1.57 (1.06-2.34)	1.54 (0.94-2.51)	1.70 (0.86-3.34)
≥40.0	3.07 (1.95-4.83)	3.60 (2.07-6.24)	2.38 (1.08-5.23)
Systolic blood pressure, mm Hg			
≤120	1 [Reference]	1 [Reference]	1 [Reference]
≥160	0.66 (0.20-2.20)	0.64 (0.14-2.84)	0.59 (0.06-5.74)
140-159	1.16 (0.76-1.78)	0.99 (0.57-1.70)	1.63 (0.83-3.21)
130-139	1.12 (0.83-1.52)	0.95 (0.65-1.40)	1.56 (0.94-2.58)
121-129	0.90 (0.65-1.26)	0.98 (0.66-1.44)	0.73 (0.39-1.39)
Estimated glomerular filtration rate, mL/min/1.73 m^2^			
60-89	1 [Reference]	1 [Reference]	1 [Reference]
≥90	0.70 (0.51-0.97)	0.72 (0.48-1.10)	0.66 (0.40-1.09)
45-59	0.93 (0.58-1.50)	1.19 (0.69-2.07)	0.57 (0.22-1.47)
30-44	1.25 (0.67-2.32)	1.19 (0.49-2.86)	1.25 (0.50-3.15)
15-29	0.97 (0.32-2.98)	0.68 (0.09-4.91)	1.08 (0.26-4.50)
<15	0.97 (0.20-4.81)	1.23 (0.14-11.24)	0.72 (0.06-8.55)
Dialysis or transplant	0.60 (0.07-5.20)	1.47 (0.17-12.59)	NA
Hemoglobin, g/dL			
≥13.0	1 [Reference]	1 [Reference]	1 [Reference]
12.0-12.9	1.23 (0.85-1.80)	1.30 (0.82-2.07)	1.11 (0.59-2.07)
11.0-11.9	1.28 (0.76-2.17)	1.40 (0.72-2.69)	1.04 (0.43-2.50)
10.0-10.9	2.24 (1.25-4.02)	2.07 (0.94-4.53)	2.36 (0.93-6.01)
9.0-9.9	1.17 (0.40-3.38)	0.51 (0.06-4.34)	1.83 (0.53-6.35)
<9.0	1.35 (0.35-5.16)	1.86 (0.36-9.65)	0.79 (0.06-10.71)

Abbreviations: aHR, adjusted hazard ratio; BMI, body mass index (calculated as weight in kilograms divided by height in meters squared); NA, not available; VTE, venous thromboembolism.

SI conversion factor: To convert hemoglobin to grams per liter, multiply by 10.

^a^ Models were also adjusted for baseline medications: oral anticoagulants, angiotensin-converting enzyme inhibitor, angiotensin II receptor blocker, β-blocker, calcium channel blocker, diuretics, aldosterone receptor antagonist, nonaspirin antiplatelet agent, statin, and other lipid-lowering agents.

## Discussion

In this large clinical practice setting assessment of outpatients with COVID-19, the absolute risk of VTE occurring after infection was low. Although high rates of VTE have been reported among patients with COVID-19 requiring hospitalization, the risk of VTE with less severe presentations of COVID-19 has been less well described, particularly in the US. A nationwide study based in Sweden of all patients with laboratory-confirmed COVID-19 and all levels of COVID-19 severity found an absolute risk of pulmonary embolism of 0.17% in the first 30 days after diagnosis, with a higher risk during the initial presentation and among older patients.^26^ A prior US-based study focusing on hospitalized patients in the same 2 health plans observed that COVID-19 was associated with a greater than 3-fold increased risk of VTE compared with matched controls.^22^ Among ambulatory adults, a recent population-based study among patients in the UK found a higher incidence of VTE in the 30 days after COVID-19 infection compared with propensity score–matched uninfected controls (hazard ratio, 21.42).^27^ Age 55 years or older, male gender, obesity, and inherited thrombophilia were associated with post-COVID-19 VTE.

Greater understanding of VTE risk can help guide thromboprophylaxis strategies. Multiplatform randomized clinical trials have found that hospitalized patients with moderate illness due to COVID-19 may benefit from the administration of therapeutically dosed heparin anticoagulants^28^ but that therapeutic anticoagulation had no net benefit, and even perhaps could harm critically ill patients.^29^ Yet, much less is known about the optimal thromboprophylaxis strategy for people with milder presentations of COVID-19 who do not require hospitalization. Our assessment in a large, unselected group of community-dwelling outpatients diagnosed with COVID-19 found that the overall rate of VTE was low, at 0.26 per 100 person-years.

There are no established evidence-based thresholds for when to administer thromboprophylaxis in outpatients with COVID-19. Outside of COVID-19, randomized clinical trials have tested whether pharmacologic prophylaxis is warranted in patients with an elevated Khorana score that corresponds to an approximate 11% predicted VTE risk over 6 months.^30,31^ For hospitalized patients, other risk scores, such as the Padua risk score and the IMPROVE risk score, help identify patients with a high enough risk of VTE to recommend inpatient or postdischarge pharmacologic VTE prophylaxis.^32,33,34^ These risk scores recommend prophylaxis when the rate of VTE exceeds 4 per 100 person-years. The rate of VTE associated with COVID-19 in unselected outpatients in our study is much lower than these thresholds for prophylaxis. For further context, the absolute rates of VTE associated with COVID-19 are not much higher than the average rate of VTE in the general population, which has been reported to be approximately 0.1 to 0.2 per 100 person-years.^35^

The low VTE rate found in our study may not justify a VTE prevention strategy of routine administration of anticoagulation given the associated costs, inconvenience, and bleeding risks in the average nonhospitalized person with COVID-19. However, we identified several subgroups of patients with VTE rates that reached or exceeded the threshold of 4 per 100 person-years, which is when more intensive VTE prevention should be considered. Specifically, the rate of VTE among people with a history of VTE was 12.44 per 100 person-years in the first 30 days after COVID-19 diagnosis and 3.76 per 100 person-years after 30 days. In the first 30 days after COVID-19, people with a history of primary or secondary thrombophilia had a VTE rate of 4.16 per 100 person-years and people who were aged 75 to 84 years had a VTE rate of 3.96 per 100 person-years.

Several randomized clinical trials have attempted to clarify the optimal use of antithrombotic agents in patients with COVID-19. Heparin-based therapeutic anticoagulants seem to improve clinical outcomes in non–critically ill hospitalized patients with COVID-19.^28,29,36,37^ A randomized clinical trial of prehospitalization antithrombotic treatment comparing 2 doses of apixaban, aspirin, and placebo was stopped due to low event rates after enrolling 657 patients.^15^ Postdischarge prophylaxis for patients hospitalized with COVID-19 who had high IMPROVE risk scores for VTE was also evaluated in a recent placebo-controlled randomized clinical trial in which prophylactic-dose rivaroxaban was associated with a lower risk of a composite outcome of thrombosis and death without an excess risk of major bleeding.^38^ Current American Society of Hematology guidelines do not recommend the routine use of anticoagulants in outpatients with COVID-19 or in those who have been recently hospitalized.^5^ Although the low rates of VTE reported in our study do not support the universal use of antithrombotic agents in nonhospitalized patients with COVID-19, our results support the importance of clinical trials that are evaluating whether some higher-risk subgroups may benefit.^39,40,41^

### Strengths and Limitations

Our study was conducted in a diverse cohort in an integrated system with longitudinal follow-up. To identify validated acute VTE, we used both claims-based approaches and a validated natural language processing algorithm applied to semistructured and unstructured EHR data. We used models that incorporate the competing risk of death, which more accurately reflects the excess risk of COVID-19–associated VTE. In addition, we identified readily available risk factors that were independently associated with VTE in outpatients with COVID-19 and subgroups of patients in whom the absolute rate of VTE approached levels at which point more intensive VTE prevention strategies might be considered. Our study was also conducted before the widespread availability of vaccines, the emergence of the Delta and Omicron variants, the development of effective outpatient therapies, and easily available home-based testing. With the advent of home-based testing, it will be more difficult to ascertain the true population-based risk of COVID-19–associated VTE, as many cases are no longer diagnosed in health care settings.

There are limitations to our study. Although the time period of our study helped reduce potential confounding due to nonrandom uptake of vaccines and expanded diagnostics and therapeutics, we acknowledge that the VTE rates in our study reflect the outcomes associated with older variants of the SARS-CoV-2 virus. The Omicron lineage and its subvariants appear to have increased immune evasion but lower clinical severity; whether VTE risk is different with Omicron is unknown. The time frame of the study also does not allow us to comment on the potential impact of vaccines on VTE risk in COVID-19. Incorporating the competing risk of death and health care plan disenrollment into our models could have led to an underestimate of VTE risk, as some deaths could have been due to undiagnosed VTE. Our study described VTE rates only in patients with COVID-19 and did not include a control group of noninfected patients; hence, we cannot comment on whether COVID-19 increases the risk of VTE in outpatients, unlike in our prior analysis of hospitalized patients.^22^ Although we attempted to include many clinical risk factors for VTE, the study cohort lacked risk factors such as hormone therapy, recent surgery, or immobilization. In addition, we collected only baseline medical conditions and did not have updated risk factors after the COVID-19 diagnosis and during the follow-up period. Additionally, although our study population is representative of the diverse population of California, this may not reflect the characteristics of other regions or health systems.

## Conclusions

In this cohort study of outpatient adults with COVID-19, we found that although the absolute risk of VTE was low overall, selected patient characteristics were associated with higher rates of VTE, particularly in the first 30 days after COVID-19 diagnosis. These findings may help identify subsets of patients with COVID-19 who could benefit from VTE preventive strategies and more intensive short-term surveillance.

## Notes

Supplement 1. **eTable.** *ICD-10* Codes for Venous Thromboembolism

Supplement 2.
Data Sharing Statement
