# Supplementary material for: Assessment of the Risk of Venous Thromboembolism in Nonhospitalized Patients With COVID-19
Source: JAMA Netw Open. 2023 Mar 13;6(3):e232338. doi: 10.1001/jamanetworkopen.2023.2338 (PMC10011935; doi:10.1001/jamanetworkopen.2023.2338)
Supplement: Supplement 1. — eTable. ICD-10 Codes for Venous Thromboembolism [file jamanetwopen-e232338-s001.pdf]

Supplementary Online Content

Fang MC, Reynolds K, Tabada GH, et al. Assessment of the risk of venous thromboembolism in nonhospitalized patients with COVID-19. *JAMA Netw Open*. 2023;6(3):e232338. doi:10.1001/jamanetworkopen.2023.2338

eTable. *ICD-10* Codes for Venous Thromboembolism

This supplementary material has been provided by the authors to give readers additional information about their work.

© 2023 Fang MC et al. *JAMA Network Open.*

**eTable.** ***ICD-10*** **codes for Venous Thromboembolism**
I26
I26.0
I26.02
I26.09
I26.9
I26.92
I26.99
I27.82
I80
I80.0
I80.00
I80.01
I80.02
I80.03
I80.1
I80.10
I80.11
I80.12
I80.13
I80.2
I80.20
I80.201
I80.202
I80.203
I80.209
I80.21
I80.211
I80.212
I80.213
I80.219
I80.22
I80.221
I80.222
I80.223
I80.229
I80.23
I80.231
I80.232
I80.233
I80.239

© 2023 Fang MC et al. *JAMA Network Open.*

I80.29
I80.291
I80.292
I80.293
I80.299
I80.3
I80.8
I80.9
I81
I82
I82.0
I82.1
I82.2
I82.21
I82.210
I82.211
I82.22
I82.220
I82.221
I82.29
I82.290
I82.291
I82.3
I82.4
I82.40
I82.401
I82.402
I82.403
I82.409
I82.41
I82.411
I82.412
I82.413
I82.419
I82.42
I82.421
I82.422
I82.423
I82.429
I82.43
I82.431
I82.432
I82.433

© 2023 Fang MC et al. *JAMA Network Open.*

I82.439
I82.44
I82.441
I82.442
I82.443
I82.449
I82.49
I82.491
I82.492
I82.493
I82.499
I82.4Y1
I82.4Y2
I82.4Y3
I82.4Y9
I82.4Z1
I82.4Z2
I82.4Z3
I82.4Z9
I82.5
I82.50
I82.501
I82.502
I82.503
I82.509
I82.51
I82.511
I82.512
I82.513
I82.519
I82.52
I82.521
I82.522
I82.523
I82.529
I82.53
I82.531
I82.532
I82.533
I82.539
I82.54
I82.541
I82.542

© 2023 Fang MC et al. *JAMA Network Open.*

I82.543
I82.549
I82.59
I82.591
I82.592
I82.593
I82.599
I82.5Y1
I82.5Y2
I82.5Y3
I82.5Y9
I82.5Z1
I82.5Z2
I82.5Z3
I82.5Z9
I82.6
I82.60
I82.601
I82.602
I82.603
I82.609
I82.61
I82.611
I82.612
I82.613
I82.619
I82.62
I82.621
I82.622
I82.623
I82.629
I82.7
I82.70
I82.701
I82.702
I82.703
I82.709
I82.71
I82.711
I82.712
I82.713
I82.719
I82.72

© 2023 Fang MC et al. *JAMA Network Open.*

I82.721
I82.722
I82.723
I82.729
I82.8
I82.81
I82.811
I82.812
I82.813
I82.819
I82.89
I82.890
I82.891
I82.9
I82.90
I82.91
I82.A
I82.A1
I82.A11
I82.A12
I82.A13
I82.A19
I82.A2
I82.A21
I82.A22
I82.A23
I82.A29
I82.B
I82.B1
I82.B11
I82.B12
I82.B13
I82.B19
I82.B2
I82.B21
I82.B22
I82.B23
I82.B29
I82.C
I82.C1
I82.C11
I82.C12
I82.C13

© 2023 Fang MC et al. *JAMA Network Open.*

I82.C19
I82.C2
I82.C21
I82.C22
I82.C23
I82.C29
K75.1

© 2023 Fang MC et al. *JAMA Network Open.*
