# Supplementary material for: Assessment of the Risk of Venous Thromboembolism in Nonhospitalized Patients With COVID-19
Source: JAMA Netw Open. 2023 Mar 13;6(3):e232338. doi: 10.1001/jamanetworkopen.2023.2338 (PMC10011935; doi:10.1001/jamanetworkopen.2023.2338)
Supplement: Supplement 2. — Data Sharing Statement [file jamanetwopen-e232338-s002.pdf]

Data Sharing Statement

Fang. Assessment of the Risk of Venous Thromboembolism in Nonhospitalized Patients With COVID-19. *JAMA Netw Open*. Published March 13, 2023.
doi:10.1001/jamanetworkopen.2023.2338

Data
Data available: No
